# Association Between Birth Weight and Prevalence of Cardiovascular Disease and Other Lifestyle-related Diseases Among the Japanese Population: The JPHC-NEXT Study

**DOI:** 10.2188/jea.JE20230045

**Published:** 2024-07-05

**Authors:** Keisuke Yoshii, Naho Morisaki, Aurélie Piedvache, Shinya Nakada, Kazuhiko Arima, Kiyoshi Aoyagi, Hiroki Nakashima, Nobufumi Yasuda, Isao Muraki, Kazumasa Yamagishi, Isao Saito, Tadahiro Kato, Kozo Tanno, Taiki Yamaji, Motoki Iwasaki, Manami Inoue, Shoichiro Tsugane, Norie Sawada

**Affiliations:** 1 Division of Endocrinology and Metabolism, National Center for Child Health and Development, Tokyo, Japan; 2 Department of Social Medicine, National Center for Child Health and Development, Tokyo, Japan; 3 Department of Public Health, Nagasaki University Graduate School of Biomedical Sciences, Nagasaki, Japan; 4 Department of Public Health, Kochi University Medical School, Kohasu, Kochi, Japan; 5 Public Health, Department of Social Medicine, Graduate School of Medicine, Osaka University, Osaka, Japan; 6 Department of Public Health Medicine, Faculty of Medicine, and Health Services Research and Development Center, University of Tsukuba, Tsukuba, Japan; 7 Ibaraki Western Medical Center, Ibaraki, Japan; 8 Department of Public Health and Epidemiology, Faculty of Medicine, Oita University, Oita, Japan; 9 Division of Life Span Development and Clinical Psychology, Graduate School of Education, Ehime University, Matsuyama, Japan; 10 Department of Hygiene and Preventive Medicine, School of Medicine, Iwate Medical University, Iwate, Japan; 11 Division of Epidemiology, National Cancer Center Institute for Cancer Control, Tokyo, Japan; 12 Division of Cohort Research, National Cancer Center Institute for Cancer Control, Tokyo, Japan; 13 Division of Prevention, National Cancer Center Institute for Cancer Control, Tokyo, Japan; 14 National Institute of Health and Nutrition, National Institutes of Biomedical Innovation, Health and Nutrition, Osaka, Japan

**Keywords:** low birth weight, cardiovascular disease, diabetes, hypertension, DoHAD

## Abstract

**Background:**

An association between birth weight and cardiovascular disease (CVD) in adulthood has been observed in many countries; however, only a few studies have been conducted in Asian populations.

**Methods:**

We used data from the baseline survey (2011–2016) of the Japan Public Health Center-based Prospective Study for the Next Generation Cohort, which included 114,105 participants aged 40–74 years. Adjusted prevalence ratios (aPRs) and 95% confidence intervals (CIs) were calculated from the prevalence of present and past histories of CVD and other lifestyle-related diseases, including hypertension, diabetes, hyperlipidemia, and gout, by birth weight, using Poisson regression.

**Results:**

The prevalence of CVD increased with lower birth weight, with the highest prevalence among those with birth weight under 1,500 grams (males 4.6%; females 1.7%) and the lowest one among those with birth weight at or over 4,000 g (males 3.7%: females 0.8%). Among 88,653 participants (41,156 males and 47,497 females) with complete data on possible confounders, birth weight under 1,500 g was associated with a higher prevalence of CVD (aPR 1.76; 95% CI, 1.37–2.26), hypertension (aPR 1.29; 95% CI, 1.17–1.42), and diabetes (aPR 1.53; 95% CI, 1.26–1.86) when a birth weight of 3,000–3,999 grams was used as the reference. Weaker associations were observed for birth weight of 1,500–2,499 grams and 2,500–2,999 grams, while no significant associations were observed for birth weight at or over 4,000 grams. The association between birth weight and the prevalence of hyperlipidemia was less profound, and no significant association was observed between birth weight and gout.

**Conclusion:**

Lower birth weight was associated with a higher prevalence of CVD, hypertension, and diabetes in the Japanese population.

## INTRODUCTION

Over the last 2 decades in Japan, the proportion of low birth weight (LBW) infants has remained in the mid-9% range, the highest among high-income countries, following a doubling from 1980 to 2000.^1^^,^^2^ As the generation born after the 1980s approaches late middle age, there is a great deal of concern regarding their development of lifestyle-related diseases because epidemiological evidence shows that adults born with LBW are more susceptible to cardiovascular disease (CVD) and its risk factors, such as hypertension and type 2 diabetes.^3^

Statistics show that the average birth weight differs by over 300 grams (g) between Japanese and Caucasians, and the prevalence of CVD and its risk factors varies largely by culture and ethnic background,^4^^,^^5^ highlighting the importance of understanding the association between birth weight and CVD risk factors in each population. While a worldwide meta-analysis has shown that LBW increases the risk of CVD, hypertension, and type 2 diabetes,^3^ most of the evidence used in the meta-analysis was provided by the non-Asian population, with only two relatively small studies from India (517 adults) and China (2,033 adults), and none from Japan regarding CVD.^6^^,^^7^ However, we found a past Japanese study on 26,949 female nurses regarding birth weight and diabetes and several local studies about birth weight and hypertension, most of which targeted adolescents or young adults.^8^^–^^14^

The association between birth weight and other lifestyle-related diseases, such as hyperlipidemia and gout, has not been well studied. Two systematic reviews suggested a limited association between birth weight and blood cholesterol levels.^15^^,^^16^ Furthermore, we failed to identify systematic reviews on birth weight, uric acid levels, or gout. Only two observational studies were found: one in 5,390 adolescents reported that LBW was associated with increased uric acid levels, and the other reported that being born small for gestational age increased the risk for gout during adulthood.^17^^,^^18^

Thus, using data from a large population-based cohort in Japan, we examined the relationship between birth weight and CVD, hypertension, diabetes, hyperlipidemia, and gout.

## METHODS

### Study population

This study is based on the Japan Public Health Center-based Prospective Study for the Next Generation (JPHC-NEXT) launched in 2011, a large-scale populational cohort consisting of residents of 16 municipalities in seven prefectures across the country, aiming to elucidate the risk and protective factors for lifestyle-related diseases and to contribute to the development of personalized healthcare.^19^ In brief, in this study, a self-administered questionnaire, which mainly asked about lifestyle factors, was distributed to all registered residents aged 40–74 years. Written informed consent was obtained at the time of recruitment to participate in a long-term follow-up of at least 20 years, as well as the potential use of their residence registry, medical records, medical fee receipts, and care insurance, in addition to the provision of biospecimens and their genomic analysis. For this study, we used data from the baseline survey, whose data collection was conducted from 2011 to 2016. This survey contained questions on birth weight, medical history, and lifestyle and was completed by 114,105 males and females (44% of the target population). The JPHC-NEXT study protocol was approved by the Institutional Review Board of the National Cancer Center (No. 2011-186) as well as at each collaborating institution in the regional areas. The analysis protocol for this study was approved by the Institutional Review boards of the National Cancer Center (No. 2017-250) as well as the National Center for Child Health and Development on June 4, 2018 (No. 1847).

### Outcomes

The outcome variables were the prevalence of present and past histories of CVD (myocardial infarction, angina, and stroke), three risk factors (hypertension, diabetes, and hyperlipidemia), and gout. The prevalence of CVD was calculated based on the following question: “Have you ever been told by a doctor that you have the following diseases (myocardial infarction, angina, or stroke [brain hemorrhage, brain infarction, or subarachnoid hemorrhage])?” and “Have you ever had a heart bypass surgery?” The prevalence of hypertension, diabetes, hyperlipidemia, and gout was calculated based on the following questions: “Have you ever been told by a doctor that you have the following diseases (hypertension, diabetes, hypercholesteremia [hyperlipidemia or dyslipidemia], and gout)?” or “Are there any medicines [antihypertensive, antidiabetic, anti-cholesterol, and antigout agent] prescribed by a doctor that you take regularly?”

### Independent variables

The variable of interest was birth weight. In the baseline survey, participants ticked one of the following categories: <1,500 g; 1,500–2,499 g; 2,500–2,999 g; 3,000–3,999 g; ≥4,000 g; and “do not know.” Furthermore, we considered the following variables as basic characteristics, including known risk factors: place of residence, age, the number of elder siblings (none; one; more than one), body mass index (BMI) at 20 years old and at the baseline survey (<18.5, 18.5–24.9, or ≥25.0 kg/m^2^), height at the baseline survey (split into quartile according to gender), parental familial history of CVD, hypertension, and diabetes, passive smoking around 10 years old (almost none; 1–3 times a month; 1–4 times a week; and almost every day), smoking status (current and former smoking), and educational attainment (junior high school; high school; junior college/specialty/college or university dropout; and college/university/graduate school). BMI at 20 years old and at the baseline survey was calculated by dividing the weight in kilograms around 20 years old and at the baseline survey by height in meters squared at the baseline survey, respectively.

### Statistical analysis

The inclusion criteria for this study were participants from the JPHC-NEXT study, while the exclusion criteria comprised participants with missing birth weight data. We further named the dataset of participants with data on all covariables for the main analysis (place of residence, age, number of older siblings, height, parental familial history, passive smoking, smoking status, and educational attainment) the complete case dataset.

In the JPHC-NEXT study, which included 114,105 participants (52,566 males and 61,539 females) who answered the baseline questionnaire, the proportions of missing data for birth weight, passive smoking, BMI at 20 years of age, educational attainment, and height at the baseline survey were 16.1%, 7.7%, 6.5%, 2.2%, and 0.6%, respectively. Notably, birth weights were missing for 18,368 participants; thus, we based our analysis on the remaining 44,079 males and 51,658 females. Finally, the complete case dataset comprised 41,156 males and 47,497 females.

For descriptive statistics, categorical variables were presented as numbers and percentages within the overall population. The χ2 test was employed to compare the prevalence of outcomes across five categories of birth weight and among those with missing birth weight information. Additionally, the Bonferroni correction was applied when interpreting pairwise comparisons conducted among multiple groups. Next, we investigated the association between each outcome and birth weight, adjusted for potential confounders, by the prevalence ratio derived from generalized linear models using Poisson regression with robust standard errors.^20^^–^^22^ Place of residence was incorporated as a fixed effect in each model. The base model, referred to as model 1, was adjusted for age and birth year to account for birth cohort effects. In the subsequent model, model 2, additional variables known to be strongly correlated with either birth weight or the risk of CVD—specifically, the number of older siblings, height at recruitment, parental familial history, passive smoking at approximately 10 years of age, and educational attainment—were included as potential confounders.^23^^,^^24^ All analyses were conducted for the overall population as well as by gender; for analysis using the overall population, all analyses were adjusted for sex, and whether effect modification by sex existed was assessed using the *P*-value for the interaction term sex × birth weight category.

The main analysis was based on a complete case dataset. As a first sensitivity analysis, we repeated our analysis on data where missing data on all the variables except the birth weight category were imputed by a chained equation to create 25 imputed data sets, which were stratified by sex for sex-specific analyses and overall for the overall analysis. For this sensitivity analysis, we additionally created two models that included smoking status and BMI at 20 years of age, which were considered as mediators due to their temporality (occurring after birth weight was determined), to check whether including these potential confounding variables influenced the results (models 3a and 3b, respectively).

Our main analysis was based on medical history, defined as having either a present or past diagnosis and/or currently taking medication. Thus, a second sensitivity analysis was conducted only for participants currently taking medications for hypertension, diabetes, hyperlipidemia, and hyperuricemia. Finally, we performed a third sensitivity analysis with those having CVD, excluding angina.

All analyses were performed using the Stata Statistical Software Release 15 (StataCorp, College Station, TX, USA). An overall view of the populations used in the analysis is shown in eFigure 1.

## RESULTS

The characteristics of the participants by birth weight category are presented in Table 1 and Table 2 for males and females, respectively. In summary, those born smaller were more likely to be older and shorter, less likely to have higher educational attainment, and more likely to have a family history of CVD. Those born at ≥4,000 g as well as those born at <1,500 g, were more likely to have a BMI of 25 kg/m^2^ or over at 20 years of age. The proportion of those with BMI <18.5 kg/m^2^ at 20 years of age and at baseline was higher among the lower birth weight categories. Finally, females were more likely to be in the lower birth weight category than males.

**Table 1.** Characteristics in male population (*n* = 52,566)—complete cases dataset

	Total	Missing on birth weight data		Male with birth weight data	Birth weight									

					<1,500 g		1,500–2,499 g		2,500–2,999 g		3,000–3,999 g		≥4,000 g	
**Total, *n* (%)**	52,566	8,487	(16.2)	44,079	437	(1.0)	4,881	(11.1)	23,708	(53.8)	14,570	(33.1)	483	(1.1)
**Age**														
40–49 years, *n* (%)	11,119	528	(6.2)	10,591	79	(18.1)	959	(19.6)	4,396	(18.5)	4,920	(33.8)	237	(49.1)
50–59 years, *n* (%)	14,423	1,391	(16.4)	13,032	122	(27.9)	1,240	(25.4)	6,666	(28.1)	4,854	(33.3)	150	(31.1)
60–69 years, *n* (%)	19,589	4,240	(50.0)	15,349	172	(39.4)	1,997	(40.9)	9,535	(40.2)	3,571	(24.5)	74	(15.3)
70–74 years, *n* (%)	7,435	2,328	(27.4)	5,107	64	(14.6)	685	(14.0)	3,111	(13.1)	1,225	(8.4)	22	(4.6)
**Having elder siblings**														
None, *n* (%)	20,539	2,985	(35.2)	17,554	207	(47.4)	2,100	(43.0)	9,193	(38.8)	5,886	(40.4)	168	(34.8)
At least one, *n* (%)	15,480	2,120	(25.0)	13,360	108	(24.7)	1,242	(25.4)	6,997	(29.5)	4,833	(33.2)	180	(37.3)
More than one, *n* (%)	16,547	3,382	(39.8)	13,165	122	(27.9)	1,539	(31.5)	7,518	(31.7)	3,851	(26.4)	135	(28.0)
**BMI at 20 years old**														
<18.5, *n* (%)	3,217	410	(4.8)	2,807	37	(8.5)	414	(8.5)	1,539	(6.5)	803	(5.5)	14	(2.9)
18.5–24.9, *n* (%)	41,959	5,993	(70.6)	35,966	316	(72.3)	3,945	(80.8)	19,659	(82.9)	11,696	(80.3)	350	(72.5)
≥25.0, *n* (%)	3,574	490	(5.8)	3,084	39	(8.9)	301	(6.2)	1,525	(6.4)	1,165	(8.0)	54	(11.2)
Missing, *n* (%)	3,816	1,594	(18.8)	2,222	45	(10.3)	221	(4.5)	985	(4.2)	906	(6.2)	65	(13.5)
**BMI at the baseline survey**														
<18.5, *n* (%)	1,500	286	(3.4)	1,214	18	(4.1)	202	(4.1)	695	(2.9)	290	(2.0)	9	(1.9)
18.5–24.9, *n* (%)	34,197	5,667	(66.8)	28,530	270	(61.8)	3,317	(68.0)	15,742	(66.4)	8,945	(61.4)	256	(53.0)
≥25.0, *n* (%)	16,056	2,201	(25.9)	13,855	131	(30.0)	1,308	(26.8)	7,068	(29.8)	5,145	(35.3)	203	(42.0)
Missing, *n* (%)	813	333	(3.9)	480	18	(4.1)	54	(1.1)	203	(0.9)	190	(1.3)	15	(3.1)
**Height at the baseline survey^a^**														
Q1, *n* (%)	13,264	3,275	(38.6)	9,989	181	(41.4)	1,734	(35.5)	6,158	(26.0)	1,897	(13.0)	19	(3.9)
Q2, *n* (%)	15,587	2,614	(30.8)	12,973	109	(24.9)	1,546	(31.7)	7,576	(32.0)	3,678	(25.2)	64	(13.3)
Q3, *n* (%)	11,838	1,443	(17.0)	10,395	82	(18.8)	921	(18.9)	5,370	(22.7)	3,911	(26.8)	111	(23.0)
Q4, *n* (%)	11,619	996	(11.7)	10,623	59	(13.5)	669	(13.7)	4,542	(19.2)	5,065	(34.8)	288	(59.6)
Missing, *n* (%)	258	159	(1.9)	99	6	(1.4)	11	(0.2)	62	(0.3)	19	(0.1)	1	(0.2)
**Familial history**														
Diabetes, *n* (%)	7,808	978	(11.5)	6,830	76	(17.4)	756	(15.5)	3,536	(14.9)	2,357	(16.2)	105	(21.7)
Hypertension, *n* (%)	15,218	2,046	(24.1)	13,172	108	(24.7)	1,460	(29.9)	6,876	(29.0)	4,571	(31.4)	157	(32.5)
Cardiovascular disease, *n* (%)	13,223	2,148	(25.3)	11,075	121	(27.7)	1,233	(25.3)	6,148	(25.9)	3,474	(23.8)	99	(20.5)
**Passive smoking around 10 years old**														
Almost none, *n* (%)	23,413	3,584	(42.2)	19,829	184	(42.1)	2,165	(44.4)	10,774	(45.4)	6,485	(44.5)	221	(45.8)
1–3 times a month, *n* (%)	2,921	371	(4.4)	2,550	25	(5.7)	289	(5.9)	1,369	(5.8)	841	(5.8)	26	(5.4)
1–4 times a week, *n* (%)	4,782	539	(6.4)	4,243	45	(10.3)	471	(9.6)	2,247	(9.5)	1,426	(9.8)	54	(11.2)
Almost every day, *n* (%)	18,053	2,787	(32.8)	15,266	134	(30.7)	1,647	(33.7)	8,058	(34.0)	5,260	(36.1)	167	(34.6)
Missing, *n* (%)	3,397	1,206	(14.2)	2,191	49	(11.2)	309	(6.3)	1,260	(5.3)	558	(3.8)	15	(3.1)
**Smoking states**														
Current and former smoking, *n* (%)	42,342	6,703	(79.0)	35,639	352	(80.5)	3,917	(80.2)	19,301	(81.4)	11,682	(80.2)	387	(80.1)
**Educational attainment**														
Junior high school, *n* (%)	10,140	3,006	(35.4)	7,134	125	(28.6)	1,019	(20.9)	4,342	(18.3)	1,605	(11.0)	43	(8.9)
High school, *n* (%)	26,028	3,711	(43.7)	22,317	210	(48.1)	2,399	(49.1)	12,252	(51.7)	7,225	(49.6)	231	(47.8)
Junior college/specialty/ ​ college or university dropout, *n* (%)	6,676	669	(7.9)	6,007	50	(11.4)	659	(13.5)	2,988	(12.6)	2,231	(15.3)	79	(16.4)
College/university/ ​ graduate school, *n* (%)	8,550	686	(8.1)	7,864	36	(8.2)	709	(14.5)	3,688	(15.6)	3,306	(22.7)	125	(25.9)
Missing, *n* (%)	1,172	415	(4.9)	757	16	(3.7)	95	(1.9)	438	(1.8)	203	(1.4)	5	(1.0)

BMI, body mass index; Q, quartile.

^a^Range of height in cm for each quartile: Q1 [127, 163], Q2 [164, 168], Q3 [169, 172], Q4 [173, 200].

**Table 2.** Characteristics in female population (*n* = 61,539)—complete cases dataset

	Total	Missing on birth weight data		Female with birth weight data	Birth weight									

					<1,500 g		1,500–2,499 g		2,500–2,999 g		3,000–3,999 g		≥4,000 g	
**Total, *n* (%)**	61,539	9,881	(16.1)	51,658	426	(0.7)	5,764	(9.4)	28,690	(46.6)	16,401	(26.7)	377	(0.6)
**Age**														
40–49 years, *n* (%)	13,362	284	(2.9)	13,078	59	(13.8)	1,124	(19.5)	5,311	(18.5)	6,365	(38.8)	219	(58.1)
50–59 years, *n* (%)	17,121	1,340	(13.6)	15,781	109	(25.6)	1,438	(24.9)	8,520	(29.7)	5,615	(34.2)	99	(26.3)
60–69 years, *n* (%)	22,130	5,126	(51.9)	17,004	182	(42.7)	2,352	(40.8)	11,092	(38.7)	3,338	(20.4)	40	(10.6)
70–74 years, *n* (%)	8,926	3,131	(31.7)	5,795	76	(17.8)	850	(14.7)	3,767	(13.1)	1,083	(6.6)	19	(5.0)
**Having elder siblings**														
None, *n* (%)	22,269	2,758	(27.9)	19,511	153	(35.9)	2,307	(40.0)	10,578	(36.9)	6,337	(38.6)	136	(36.1)
At least one, *n* (%)	17,796	2,347	(23.8)	15,449	115	(27.0)	1,522	(26.4)	8,124	(28.3)	5,539	(33.8)	149	(39.5)
More than one, *n* (%)	21,474	4,776	(48.3)	16,698	158	(37.1)	1,935	(33.6)	9,988	(34.8)	4,525	(27.6)	92	(24.4)
**BMI at 20 years old**														
<18.5, *n* (%)	7,492	768	(7.8)	6,724	73	(17.1)	953	(16.5)	3,674	(12.8)	1,983	(12.1)	41	(10.9)
18.5–24.9, *n* (%)	46,919	6,459	(65.4)	40,460	304	(71.4)	4,289	(74.4)	22,693	(79.1)	12,883	(78.6)	291	(77.2)
≥25.0, *n* (%)	3,559	685	(6.9)	2,874	31	(7.3)	339	(5.9)	1,483	(5.2)	996	(6.1)	25	(6.6)
Missing, *n* (%)	3,569	1,969	(19.9)	1,600	18	(4.2)	183	(3.2)	840	(2.9)	539	(3.3)	20	(5.3)
**BMI at the baseline survey**														
<18.5, *n* (%)	4,558	616	(6.2)	3,942	37	(8.7)	527	(9.1)	2,214	(7.7)	1,137	(6.9)	27	(7.2)
18.5–24.9, *n* (%)	42,179	6,295	(63.7)	35,884	266	(62.4)	3,956	(68.6)	20,119	(70.1)	11,297	(68.9)	246	(65.3)
≥25.0, *n* (%)	13,502	2,461	(24.9)	11,041	117	(27.5)	1,184	(20.5)	5,979	(20.8)	3,670	(22.4)	91	(24.1)
Missing, *n* (%)	1,300	509	(5.2)	791	6	(1.4)	97	(1.7)	378	(1.3)	297	(1.8)	13	(3.4)
**Height at the baseline survey^a^**														
Q1, *n* (%)	15,322	3,929	(39.8)	11,393	180	(42.3)	2,101	(36.5)	7,310	(25.5)	1,784	(10.9)	18	(4.8)
Q2, *n* (%)	18,345	3,035	(30.7)	15,310	143	(33.6)	1,779	(30.9)	9,261	(32.3)	4,076	(24.9)	51	(13.5)
Q3, *n* (%)	14,446	1,672	(16.9)	12,774	62	(14.6)	1,108	(19.2)	6,847	(23.9)	4,647	(28.3)	110	(29.2)
Q4, *n* (%)	12,960	968	(9.8)	11,992	39	(9.2)	740	(12.8)	5,170	(18.0)	5,845	(35.6)	198	(52.5)
Missing, *n* (%)	466	277	(2.8)	189	2	(0.5)	36	(0.6)	102	(0.4)	49	(0.3)	0	(0.0)
**Familial history**														
Diabetes, *n* (%)	9,534	1,079	(10.9)	8,455	69	(16.2)	924	(16.0)	4,394	(15.3)	2,969	(18.1)	99	(26.3)
Hypertension, *n* (%)	20,605	2,491	(25.2)	18,114	121	(28.4)	1,975	(34.3)	9,823	(34.2)	6,051	(36.9)	144	(38.2)
Cardiovascular disease, *n* (%)	16,489	2,609	(26.4)	13,880	129	(30.3)	1,633	(28.3)	7,985	(27.8)	4,040	(24.6)	93	(24.7)
**Passive smoking around 10 years old**														
Almost none, *n* (%)	28,645	4,488	(45.4)	24,157	185	(43.4)	2,670	(46.3)	13,757	(48.0)	7,385	(45.0)	160	(42.4)
1–3 times a month, *n* (%)	2,310	207	(2.1)	2,103	8	(1.9)	225	(3.9)	1,154	(4.0)	693	(4.2)	23	(6.1)
1–4 times a week, *n* (%)	4,586	424	(4.3)	4,162	33	(7.7)	378	(6.6)	2,297	(8.0)	1,424	(8.7)	30	(8.0)
Almost every day, *n* (%)	20,644	2,827	(28.6)	17,817	140	(32.9)	2,006	(34.8)	9,428	(32.9)	6,094	(37.2)	149	(39.5)
Missing, *n* (%)	5,354	1,935	(19.6)	3,419	60	(14.1)	485	(8.4)	2,054	(7.2)	805	(4.9)	15	(4.0)
**Smoking status, *n* (%)**														
Current and former smoking, *n* (%)	10,042	1,019	(10.3)	9,023	81	(19.0)	996	(17.3)	4,478	(15.6)	3,377	(20.6)	91	(24.1)
**Educational attainment**														
Junior high school, *n* (%)	11,667	4,009	(40.6)	7,658	131	(30.8)	1,204	(20.9)	4,996	(17.4)	1,299	(7.9)	28	(7.4)
High school, *n* (%)	30,599	4,134	(41.8)	26,465	198	(46.5)	2,916	(50.6)	14,925	(52.0)	8,256	(50.3)	170	(45.1)
Junior college/specialty/ ​ college or university dropout, *n* (%)	14,787	1,102	(11.2)	13,685	69	(16.2)	1,278	(22.2)	6,891	(24.0)	5,312	(32.4)	135	(35.8)
College/university/ ​ graduate school, *n* (%)	3,207	113	(1.1)	3,094	17	(4.0)	254	(4.4)	1,415	(4.9)	1,368	(8.3)	40	(10.6)
Missing, *n* (%)	1,279	523	(5.3)	756	11	(2.6)	112	(1.9)	463	(1.6)	166	(1.0)	4	(1.1)

BMI, body mass index; Q, quartile.

^a^Range of height in cm for each quartile: Q1 [125, 150], Q2 [151, 155], Q3 [156, 159], Q4 [160, 195].

Table 3 shows the prevalence of present and past histories of CVD, hypertension, diabetes, hyperlipidemia, and gout by birth weight. Among females, a higher prevalence of CVD, hypertension, diabetes, and hyperlipidemia was observed in those with lower birth weight than in those born at 3,000–3,999 g. On the other hand, those born with a birth weight of ≥4,000 g showed a lower risk of hypertension (9.5%) compared to the reference, but no significant difference was observed for other outcomes. Among males, the prevalence of CVD, hypertension, and diabetes was also higher among those with lower birth weight, except for hyperlipidemia, which was highest among those born at 2,500–2,999 g. Moreover, no significant differences were observed for gout for either sex except when males born at 1,500–2,499 g were compared to males born at 3,000–3,999 g. The prevalence of CVD, hypertension, diabetes, and gout was higher in males, while that of diabetes was higher in females.

**Table 3.** Prevalences of cardiovascular disease and four lifestyle-related diseases based on birth weight

	Total		Birth weight^a^											

			Missing		<1,500 g		1,500–2,499 g		2,500–2,999 g		3,000–3,999 g		≥4,000 g	
**Male (*n* = 52,566)**														
Cardiovascular disease, *n* (%)	2,603	(5.9)	691	(8.1)	49	(11.2)	357	(7.3)	1,514	(6.4)	665	(4.6)	18	(3.7)
					*P* < 0.001		*P* < 0.001		*P* < 0.001		ref		*P* = 0.387	
Hypertension, *n* (%)	12,980	(29.5)	3,181	(37.5)	157	(35.9)	1,581	(32.4)	7,519	(31.7)	3,626	(24.9)	97	(20.1)
					*P* < 0.001		*P* < 0.001		*P* < 0.001		ref		*P* = 0.020	
Diabetes, *n* (%)	4,621	(10.5)	1,123	(13.2)	72	(16.5)	622	(12.7)	2,642	(11.1)	1,251	(8.6)	34	(7.0)
					*P* < 0.001		*P* < 0.001		*P* < 0.001		ref		*P* = 0.236	
Hyperlipidemia, *n* (%)	7,740	(17.6)	1,513	(17.8)	72	(16.5)	882	(18.1)	4,317	(18.2)	2,397	(16.5)	72	(14.9)
					*P* = 0.989		*P* = 0.009		*P* < 0.001		ref		*P* = 0.371	
Gout, *n* (%)	3,665	(8.3)	664	(7.8)	34	(7.8)	397	(8.1)	2,010	(8.5)	1,190	(8.2)	34	(7.0)
					*P* = 0.771		*P* = 0.940		*P* = 0.287		ref		*P* = 0.375	

**Female (*n* = 61,539)**														
Cardiovascular disease, *n* (%)	1,362	(2.6)	436	(4.4)	24	(5.6)	226	(3.9)	824	(2.9)	285	(1.7)	3	(0.8)
					*P* < 0.001		*P* < 0.001		*P* < 0.001		ref		*P* = 0.177	
Hypertension, *n* (%)	11,179	(21.6)	3,413	(34.5)	145	(34.0)	1,519	(26.4)	6,936	(24.2)	2,543	(15.5)	36	(9.5)
					*P* < 0.001		*P* < 0.001		*P* < 0.001		ref		*P* = 0.002	
Diabetes, *n* (%)	2,576	(5.0)	878	(8.9)	44	(10.3)	394	(6.8)	1,560	(5.4)	564	(3.4)	14	(3.7)
					*P* < 0.001		*P* < 0.001		*P* < 0.001		ref		*P* = 0.772	
Hyperlipidemia, *n* (%)	10,637	(20.6)	3,107	(31.4)	105	(24.6)	1,382	(24.0)	6,588	(23.0)	2,520	(15.4)	42	(11.1)
					*P* < 0.001		*P* < 0.001		*P* < 0.001		ref		*P* = 0.028	
Gout, *n* (%)	171	(0.3)	43	(0.4)	3	(0.7)	29	(0.5)	101	(0.4)	38	(0.2)	0	(0.0)
					*P* = 0.063		*P* = 0.002		*P* = 0.028		ref		—	

^a^Numbers in parenthesis are prevalence of each disease in each group.

Table 4 shows the association between birth weight and the five outcomes using an adjusted prevalence ratio (aPR) with reference set at the category with a birth weight of 3,000–3,999 g. Significant interaction between birth weight and sex was observed for the prevalence ratio of hypertension, hyperlipidemia, and gout. Overall, the point estimates of aPR for CVD, hypertension, and diabetes were mostly higher in categories with lower birth weight after adjusting for confounders in both model 1 and model 2 and in either sex strata. Furthermore, compared to that of the reference category, birth weight below 1,500 g was associated with significantly higher prevalence of CVD (overall: aPR 1.76; 95% confidence interval [CI], 1.37–2.26, male: aPR 1.68l 95% CI, 1.23–2.28, female: aPR 1.96; 95% CI, 1.27–3.03), hypertension (overall: aPR 1.29; 95% CI, 1.17–1.42, male: aPR 1.19; 95% CI, 1.04–1.36, female: aPR 1.38; 95% CI, 1.20–1.59), and diabetes (overall: aPR 1.53; 95% CI, 1.26–1.86, male: aPR 1.49; 95% CI, 1.17–1.89, female: aPR 1.55; 95% CI, 1.11–2.18) in model 2. Similarly, birth weight at 1,500–2,499 g was associated with a significantly higher prevalence of CVD (overall: aPR 1.25; 95% CI, 1.12–1.39, male: aPR 1.16; 95% CI, 1.01–1.32, female: aPR 1.45; 95% CI, 1.20–1.75) and diabetes (overall: aPR 1.26; 95% CI, 1.16–1.36, male: aPR 1.21; 95% CI, 1.10–1.33, female: aPR 1.31; 95% CI, 1.14–1.50) and higher prevalence of hypertension in females only (aPR 1.11; 95% CI, 1.05–1.18). We failed to observe any significant associations between birth weight and hyperlipidemia or gout except for a slightly higher prevalence of hyperlipidemia among females born at 1,500–2,499 g (aPR 1.07; 95% CI, 1.01–1.14) and at 2,500–2,999 g (aPR 1.06; 95% CI, 1.01–1.10) in model 1 (adjusted for age, and birthyear), an association that was attenuated when adjusted for additional variables in model 2.

**Table 4.** Adjusted prevalence ratio in overall population (*n* = 88,653), male population (*n* = 41,156) and female population (*n* = 47,497)—complete cases dataset

		Birth weight^e^				

		<1,500 g	1,500–2,499 g	2,500–2,999 g	3,000–3,999 g	≥4,000 g
		aPR [95% CI]	aPR [95% CI]	aPR [95% CI]		aPR [95% CI]
**Cardiovascular disease**						

Pooled^d^	Model 1^a^	1.82 [1.42–2.35]^***^	1.27 [1.14–1.41]^***^	1.08 [1.00–1.17]	ref	1.03 [0.67–1.58]

	Model 2^b^	1.76 [1.37–2.26]^***^	1.25 [1.12–1.39]^***^	1.07 [0.99–1.16]	ref	1.03 [0.67–1.59]

Male	Model 1^a^	1.72 [1.26–2.34]^**^	1.18 [1.03–1.34]^*^	1.07 [0.97–1.17]	ref	1.14 [0.71–1.82]

	Model 2^b^	1.68 [1.23–2.28]^**^	1.16 [1.01–1.32]^*^	1.06 [0.97–1.17]	ref	1.15 [0.72–1.84]

Female	Model 1^a^	2.07 [1.32–3.22]^**^	1.48 [1.23–1.78]^***^	1.12 [0.97–1.29]	ref	0.64 [0.21–1.98]

	Model 2^b^	1.96 [1.27–3.03]^**^	1.45 [1.20–1.75]^***^	1.10 [0.95–1.27]	ref	0.62 [0.20–1.92]

Hypertension						

Pooled^d^	Model 1^a^	1.26 [1.14–1.39]^***^	1.09 [1.05–1.13]^***^	1.06 [1.03–1.08]^***^	ref	0.98 [0.84–1.14]

	Model 2^b^	1.29 [1.17–1.42]^***^	1.08 [1.04–1.12]^***^	1.06 [1.03–1.09]^***^	ref	0.97 [0.83–1.12]

Male	Model 1^a^	1.14 [1.00–1.31]	1.04 [0.99–1.09]	1.03 [1.00–1.07]	ref	1.00 [0.84–1.20]

	Model 2^b^	1.19 [1.04–1.36]^*^	1.04 [0.98–1.09]	1.05 [1.01–1.08]^**^	ref	0.98 [0.83–1.17]

Female	Model 1^a^	1.40 [1.21–1.62]^***^	1.15 [1.09–1.22]^***^	1.08 [1.04–1.13]^***^	ref	0.85 [0.62–1.17]

	Model 2^b^	1.38 [1.20–1.59]^***^	1.11 [1.05–1.18]^***^	1.06 [1.02–1.11]^**^	ref	0.85 [0.62–1.16]

Diabetes						

Pooled^d^	Model 1^a^	1.61 [1.32–1.96]^***^	1.27 [1.18–1.38]^***^	1.08 [1.03–1.15]^**^	ref	1.07 [0.80–1.43]

	Model 2^b^	1.53 [1.26–1.86]^***^	1.26 [1.16–1.36]^***^	1.08 [1.03–1.15]^**^	ref	0.97 [0.74–1.29]

Male	Model 1^a^	1.51 [1.18–1.91]^**^	1.22 [1.11–1.34]^***^	1.07 [1.00–1.14]	ref	0.97 [0.69–1.36]

	Model 2^b^	1.49 [1.17–1.89]^**^	1.21 [1.10–1.33]^***^	1.08 [1.01–1.15]^*^	ref	0.89 [0.64–1.23]

Female	Model 1^a^	1.79 [1.28–2.51]^**^	1.37 [1.20–1.57]^***^	1.11 [1.01–1.23]^*^	ref	1.34 [0.76–2.36]

	Model 2^b^	1.55 [1.11–2.18]^*^	1.31 [1.14–1.50]^***^	1.09 [0.98–1.21]	ref	1.18 [0.68–2.05]

Hyperlipidemia						

Pooled^d^	Model 1^a^	0.94 [0.81–1.08]	1.04 [0.99–1.09]	1.03 [1.00–1.06]	ref	1.06 [0.89–1.26]

	Model 2^b^	0.94 [0.81–1.09]	1.02 [0.98–1.07]	1.03 [1.00–1.06]	ref	1.07 [0.90–1.27]

Male	Model 1^a^	0.86 [0.68–1.09]	0.97 [0.90–1.05]	0.99 [0.94–1.04]	ref	1.02 [0.82–1.27]

	Model 2^b^	0.90 [0.71–1.13]	0.97 [0.90–1.05]	1.00 [0.95–1.05]	ref	1.02 [0.82–1.27]

Female	Model 1^a^	0.97 [0.80–1.17]	1.07 [1.01–1.14]^*^	1.06 [1.01–1.10]^*^	ref	1.02 [0.77–1.35]

	Model 2^b^	0.95 [0.78–1.15]	1.05 [0.99–1.11]	1.04 [1.00–1.09]	ref	1.03 [0.78–1.37]

**Gout**						

Pooled^d^	Model 1^a^	0.96 [0.69–1.34]	0.97 [0.86–1.08]	0.97 [0.91–1.04]	ref	0.92 [0.66–1.29]

	Model 2^b^	1.07 [0.77–1.50]	1.03 [0.92–1.15]	1.02 [0.95–1.10]	ref	0.89 [0.64–1.24]

Male	Model 1^a^	0.90 [0.64–1.28]	0.94 [0.84–1.06]	0.97 [0.91–1.05]	ref	0.94 [0.67–1.30]

	Model 2^b^	1.01 [0.72–1.43]	1.01 [0.90–1.13]	1.02 [0.95–1.10]	ref	0.90 [0.65–1.26]

Female	Model 1^a^	2.22 [0.69–7.13]	1.40 [0.83–2.34]	0.96 [0.65–1.42]	ref	^c^

	Model 2^b^	2.48 [0.77–7.94]	1.53 [0.90–2.62]	1.02 [0.69–1.52]	ref	^c^

aPR, adjusted prevalence ratio; CI, confidence interval; ref, reference.

^a^Model 1: age + birth year + gender for pooled analysis, age + birth year for gender specific analysis.

^b^Model 2: model 1 + educational attainment, family history, passive smoking around 10 years old, height at the baseline study, having elder siblings.

^c^aPR in birth weight category 4 kg and above were not calculatable among females for gout as there were no cases of gout in this group.

^d^Interaction between gender and birth weight (*P* < 0.05) was observed for hypertension, hyperlipidemia and gout but not for CVD and diabetes.

^e^*P*-values: ^*^P < 0.05, ^**^P < 0.01, ^***^P < 0.001.

As for birth weight higher than the reference (birth weight 4,000 g and higher), we failed to observe any significant associations with outcomes when adjusted for confounders, either overall or when stratified by gender. Point estimates for aPRs were slightly higher than 1 for CVD in males (aPR 1.15; 95% CI, 0.72–1.84), diabetes in females (aPR 1.18; 95% CI, 0.68–2.05), and hyperlipidemia (males: aPR 1.02; 95% CI, 0.82–1.27; females: aPR 1.03; 95% CI, 0.78–1.37), but lower than 1 for other associations.

The characteristics of the imputed datasets used in our first sensitivity analysis and how the distribution of characteristics differed from the complete case dataset are summarized in eTable 1 and eTable 2 for males and females, respectively. When this dataset was used to assess the association between birth weight and outcomes with reference to birth weight set at 3,000–4,000 g, the point estimate of aPRs and significance of the associations were very similar to the main analysis, with higher aPRs of CVD, hypertension, and diabetes observed for categories of birth weight of <1,500 g, 1,500–2,499 g, and 2,500–2,999 g in descending order in both sexes, as well as overall. Similarly, two additional models, including smoking status and BMI at 20 years of age, only slightly changed the point estimates (eTable 3 models 3 and 4, respectively).

In the second sensitivity analysis, in which we set the outcomes as current medications for hypertension, diabetes, hyperlipidemia, and gout (eTable 4), we also observed an inverse association between birth weight and hypertension and diabetes in both males and females. Furthermore, regarding hyperlipidemia, significant associations between birth weight of 1,500–2,499 g and 2,500–2,999 g were observed among females and in model 1 only. We failed to observe significant associations between birth weight categories and the prevalence of gout/hyperuricemia in either sex.

The third sensitivity analysis, where we modified the definition of the present and history of CVD by including/excluding angina, showed that this modification slightly changed the estimated aPRs, but significance did not change except for model 2 for males with a birth weight of 1,500–2,499 g which became insignificant when excluding angina (aPR 1.15; 95% CI, 0.98–1.35) (eTable 5).

## DISCUSSION

In this large population-based study in Japan, we found that being born as LBW was associated with a higher prevalence of CVD, hypertension, and diabetes during late adulthood. In contrast, we observed that the association between LBW and prevalent hyperlipidemia was less profound, and no significant association was observed for gout.

To our knowledge, this is the first and largest report on the association between birth weight and CVD in a Japanese cohort. Notably, the association first reported in England^25^^,^^26^ has been replicated among many populations and recently confirmed through a large meta-analysis where those with birth weight less than 2.5 kg showed a 1.3 times higher risk of CVD than did those born at 4–4.5 kg.^3^ However, most of the included studies were conducted among those of European ancestry, with only two small studies from Asia. In our study, adults born with a birth weight ranging from 1.5 to 2.5 kg exhibited a 1.16-fold higher prevalence of CVD in males and a 1.45-fold increase in females. Furthermore, those born with a weight below 1.5 kg demonstrated an even higher prevalence—1.69 times in males and 1.97 times in females—compared to that of those with a birth weight of 3–4 kg. These estimates were similar to those reported from the meta-analysis and a small study of 2,033 adults in China,^7^ where adults born below 3 kg had a 1.3 times higher risk of CVD than did those with birth weight 3.0–3.5 kg. Moreover, we observed a higher prevalence of hypertension and diabetes among those with LBW, in line with a recent meta-analysis and several small studies^3^^,^^9^^–^^11^

Previous studies have suggested several possible mechanisms for the increased risk of cardiovascular diseases, hypertension, and diabetes among those with LBW.^27^^,^^28^ Examples include metabolic stress amplified during critical periods of fetal growth, resulting in epigenetic changes, decreased leptin levels, reduced nephron counts, and altered intracellular insulin signaling pathways, all of which could lead to crucial disruptions to the endocrine and cardiovascular systems later in life.^3^^,^^27^^,^^28^

Notably, CVD is Japan’s second most common cause of death, with over 5 million and 15 million individuals undergoing diabetes mellitus and hypertension treatment, respectively.^29^ Concurrently, a decrease in average birth weight has been observed in Japan since the 1980s.^1^^,^^2^ Our finding that lower birth weight is associated with a higher prevalence of CVD and lifestyle-related diseases indicates that the medical burden of the population may increase as these children reach middle age, possibly from around the 2030s. Strengthening the current preventive medicine strategies, such as incorporating stratification based on previous history, including perinatal factors, may be a possible preventive strategy for these diseases and largely contribute to population health and reducing health care costs. Further research is needed to find an optimal approach for preventive medicine strategies for such diseases.

Whether there are sex-based differences in the association between birth weight and CVD risk factors remains debatable. A previous meta-analysis reported that the association between low (<2.5 kg) and high (>4.5 kg) birth weight and risk of CVD as well as type 2 diabetes was stronger among females, while no sex-specific differences were observed for hypertension.^33^ Furthermore, a preceding meta-analysis has reported that sex differences in individual studies regarding the association between birth weight and blood pressure may be chance findings rather than true differences.^30^ In our study, we observed a small but significant interaction for all outcomes except for CVD but failed to observe a significant interaction between birth weight and CVD. However, based on the findings from larger meta-analyses of other populations, replication in other studies is required before considering that effect modification by sex may exist among Japanese individuals.

This study showed that there was no significant association between birth weight of ≥4,000 g and prevalence of CVD, their three risk factors, and gout, while the previous large studies revealed that the association was U-shaped with increased risk observed not only among those with LBW but also among those with high birth weight for CVD and diabetes.^3^ One reason for this discrepancy could lie in the variations in categorization. Specifically, the meta-analysis observed a heightened risk among individuals with a birth weight exceeding 4.5 kg, using 4.0–4.5 kg as the reference category. In contrast, our analysis employed “4 kg or over” as the uppermost category for birth weight. Furthermore, birth statistics in 2020 show that currently, less than 0.1% of births in Japan have a birth weight of over 4.5 kg,^31^ thus it may be difficult to detect this increased risk among the Japanese, even if it exists.

Two systematic reviews reported that hyperlipidemia was not strongly correlated with birth weight compared to CVD, hypertension, and diabetes, with one reporting a weak relationship among adolescents^15^ and the other reporting no association among adults.^16^ In Japan, several studies have reported an inverse association between birth weight and serum total cholesterol in a small number of adolescents^11^^–^^13^; however, a previous study in adults showed a significant inverse association only among men and not women.^10^

Further, we failed to find previous studies on the association between birth weight and gout, except for two studies; one of them reported that American adolescents born with LBW had higher levels of serum uric acid,^17^ while the other from Sweden indicated that being small for gestational age increased the risk of developing gout in young adults.^18^ These studies speculated that the low nephron number observed among those born with LBW led to a delay in uric acid metabolism in the kidney, resulting in hyperuricemia and gout.^32^ However, our study, the first in the Asian population, revealed no significant association between birth weight and the prevalence of gout during late adulthood. Studies in other populations regarding the association between birth weight and gout and hyperuricemia are anticipated.

### Strengths and limitations

The main strength of this study is its large sample size, as we used data from an adult cohort that included information on birth weight in its baseline survey. Epidemiological research on the relationship between perinatal factors and diseases during late adulthood in Japan has been sparse, mainly because of the lack of large cohorts, including perinatal history, as the participants of birth cohorts, most of which were launched in the 2000s and the 2010s, are still in the childhood/adolescent period.

This study had several limitations. First, the validity of birth weight and outcomes may be limited, as they were self-reported. While a previous Japanese study suggested that self-reported birth weight categories may correlate with actual birth weight (*r* = 0.73; *P* < 0.025)^10^ in Japan, where all citizens have official documentation of birth weight in maternal and child handbooks, birth weight data collected from a self-administered questionnaire may be inaccurate. Specifically, a large population cohort study preceding the JPHC-NEXT study showed that CVD’s sensitivity and positive predictive values were 70–80% and 40–60% of those of clinical registries, respectively.^33^ However, in our study, a sensitivity analysis omitting angina from the definition of CVD showed similar findings (eTable 5).

Second, the association between birth weight and CVD was also subject to selection bias, as the study participants were limited to those who were alive and healthy enough to participate in the baseline survey. Importantly, CVD is fatal, with death within 30 days from the onset of acute myocardial infarction reported to be 9.4%^34^; moreover, those with LBW have been reported to have higher mortality in childhood and shorter life expectancy compared to the general population born with higher birth weight.^35^ Thus, we believe that selection bias (only including survivors) would have biased the results towards the null rather than away from the null, as it is likely that more people born with lower birth weight died from CVD before the baseline survey.

Third, the association between birth weight and health outcomes observed in this population (born between 1937–1977) may differ from those of more recent generations, where an increase in the prevalence of LBW is a problem. Future studies on younger generations are required.

### Conclusion

We observed a relationship between lower birth weight and a higher prevalence of CVD, hypertension, and diabetes in a large Japanese population. The association among LBW, prevalent hyperlipidemia, and gout was less profound. Our findings suggest that the decrease in birth weight in Japan since the 1980s may attenuate the improvement in the population-level health burden.

## SUPPLEMENTARY MATERIAL

eTable 1. Characteristics in male population—imputed dataset (*n* = 44,079)

eTable 2. Characteristics in female population—imputed dataset (*n* = 51,658)

eTable 3. Adjusted prevalence ratio in overall population (*n* = 95,737), in male population (*n* = 44,079) and female population (*n* = 51,658)—imputed dataset

eTable 4. Adjusted prevalence ratio in overall population (*n* = 88,653), male population (*n* = 41,156) and female population (*n* = 47,497) on medication—complete cases dataset

eTable 5. Adjusted prevalence ratio in overall population (*n* = 88,653), male population (*n* = 41,156) and female population (*n* = 47,497)—including and excluding angina

eFigure 1. Population flow chart
