# Supplementary material for: Association Between Birth Weight and Prevalence of Cardiovascular Disease and Other Lifestyle-related Diseases Among the Japanese Population: The JPHC-NEXT Study
Source: J Epidemiol. 2024 Jul 5;34(7):307–15. doi: 10.2188/jea.JE20230045 (PMC11167263; doi:10.2188/jea.JE20230045)
Supplement: Supplementary file 1 [file je-34-307-s001.pdf]

eTable 1. Characteristics in male population — imputed dataset (n=44,079)

	Birth weight[b]									
	<1,500 g		1,500–2,499 g		2,500–2,999 g		3,000–3,999 g		≥4,000 g	
	CC	Imputed	CC	Imputed	CC	Imputed	CC	Imputed	CC	Imputed
**BMI at 20 years old**										
<18.5, *n* (%)	37 (9.4)	40 (9.3)	414 (8.9)	430 (8.8)	1,539 (6.8)	1,588 (6.7)	803 (5.9)	839 (5.8)	14 (3.4)	15 (3.1)
18.5–24.9, *n* (%)	316 (80.6)	351 (80.3)	3,945 (84.7)	4,127 (84.5)	19,659 (86.5)	20,493 (86.4)	11,696 (85.6)	12,434 (85.3)	350 (83.7)	400 (82.8)
≥25.0, *n* (%)	39 (10)	46 (10.4)	301 (6.5)	324 (6.6)	1,525 (6.7)	1,626 (6.9)	1,165 (8.5)	1,297 (8.9)	54 (12.9)	68 (14.0)
**BMI at the baseline survey**										
<18.5, *n* (%)	18 (4.3)	19 (4.2)	202 (4.2)	204 (4.2)	695 (3)	700 (3.0)	290 (2)	293 (2.0)	9 (1.9)	9 (1.9)
18.5–24.9, *n* (%)	270 (64.4)	280 (64.1)	3,317 (68.7)	3,350 (68.6)	15,742 (67)	15,870 (66.9)	8,945 (62.2)	9,046 (62.1)	256 (54.7)	264 (54.7)
≥25.0, *n* (%)	131 (31.3)	138 (31.6)	1,308 (27.1)	1,327 (27.2)	7,068 (30.1)	7,138 (30.1)	5,145 (35.8)	5,231 (35.9)	203 (43.4)	210 (43.4)
**Height at the baseline survey[a]**										
Q1, *n* (%)	181 (42)	184 (42.2)	1,734 (35.6)	1,739 (35.6)	6,158 (26)	6,180 (26.1)	1,897 (13)	1,900 (13.0)	19 (3.9)	19 (4.0)
Q2, *n* (%)	109 (25.3)	110 (25.2)	1,546 (31.8)	1,550 (31.8)	7,576 (32)	7,593 (32.0)	3,678 (25.3)	3,684 (25.3)	64 (13.3)	64 (13.3)
Q3, *n* (%)	82 (19)	83 (19.0)	921 (18.9)	923 (18.9)	5,370 (22.7)	5,382 (22.7)	3,911 (26.9)	3,916 (26.9)	111 (23)	111 (23.0)
Q4, *n* (%)	59 (13.7)	59 (13.6)	669 (13.7)	670 (13.7)	4,542 (19.2)	4,552 (19.2)	5,065 (34.8)	5,070 (34.8)	288 (59.8)	288 (59.7)
**Passive smoking around 10 years old**										
Almost none, *n* (%)	184 (47.4)	208 (47.7)	2,165 (47.4)	2,321 (47.6)	10,774 (48)	11,410 (48.1)	6,485 (46.3)	6,763 (46.4)	221 (47.2)	228 (47.2)
1–3 times a month, *n* (%)	25 (6.4)	28 (6.4)	289 (6.3)	308 (6.3)	1,369 (6.1)	1,446 (6.1)	841 (6)	874 (6.0)	26 (5.6)	27 (5.6)
1–4 times a week, *n* (%)	45 (11.6)	49 (11.3)	471 (10.3)	499 (10.2)	2,247 (10)	2,371 (10.0)	1,426 (10.2)	1,482 (10.2)	54 (11.5)	55 (11.5)
Almost every day, *n* (%)	134 (34.5)	151 (34.6)	1,647 (36)	1,753 (35.9)	8,058 (35.9)	8,481 (35.8)	5,260 (37.5)	5,451 (37.4)	167 (35.7)	173 (35.8)
**Educational attainment**										
Junior high school, *n* (%)	125 (29.7)	131 (29.9)	1,019 (21.3)	1,042 (21.4)	4,342 (18.7)	4,442 (18.7)	1,605 (11.2)	1,633 (11.2)	43 (9)	46 (9.1)
High school, *n* (%)	210 (49.9)	218 (49.8)	2,399 (50.1)	2,451 (50.2)	12,252 (52.7)	12,491 (52.7)	7,225 (50.3)	7,328 (50.3)	231 (48.3)	47 (48.3)
Junior college/specialty/ college or university dropout, *n* (%)	50 (11.9)	51 (11.8)	659 (13.8)	669 (13.7)	2,988 (12.8)	3,033 (12.8)	2,231 (15.5)	2,261 (15.5)	79 (16.5)	48 (16.5)
College/university/ graduate school, *n* (%)	36 (8.6)	37 (8.5)	709 (14.8)	719 (14.7)	3,688 (15.9)	3,743 (15.8)	3,306 (23)	3,347 (23.0)	125 (26.2)	49 (26.1)

CC, complete cases; BMI, body mass index; Q, quartile.

[a] Range of height in cm for each quartile: Q1 [127,163], Q2 [164, 168], Q3 [169, 172], Q4 [173, 200].

[b] Numbers in parenthesis are percentages among total population for each column.

eTable 2. Characteristics in female population — imputed dataset (n=51,658)

	Birth weight[b]									
	<1,500 g		1,500–2,499 g		2,500–2,999 g		3,000–3,999 g		≥4,000 g	
	CC	Imputed	CC	Imputed	CC	Imputed	CC	Imputed	CC	Imputed
**BMI at 20 years old**										
<18.5, *n* (%)	73 (17.9)	75 (17.7)	953 (17.1)	982 (17.0)	3,674 (13.2)	3,771 (13.1)	1,983 (12.5)	2,044 (12.5)	41 (11.5)	43 (11.5)
18.5–24.9, *n* (%)	304 (74.5)	317 (74.4)	4,289 (76.9)	4,427 (76.8)	22,693 (81.5)	23,372 (81.5)	12,883 (81.2)	13,314 (81.2)	291 (81.5)	307 (81.4)
≥25.0, *n* (%)	31 (7.6)	33 (7.8)	339 (6.1)	355 (6.2)	1,483 (5.3)	1,547 (5.4)	996 (6.3)	1,043 (6.4)	25 (7)	27 (7.1)
**BMI at the baseline survey**										
<18.5, *n* (%)	37 (8.8)	37 (8.8)	527 (9.3)	536 (9.3)	2,214 (7.8)	2,238 (7.8)	1,137 (7.1)	1,156 (7.0)	27 (7.4)	28 (7.4)
18.5–24.9, *n* (%)	266 (63.3)	269 (63.1)	3,956 (69.8)	4,016 (69.7)	20,119 (71.1)	20,366 (71.0)	11,297 (70.2)	11,484 (70.0)	246 (67.6)	253 (67.0)
≥25.0, *n* (%)	117 (27.9)	120 (28.1)	1,184 (20.9)	1,212 (21.0)	5,979 (21.1)	6,086 (21.2)	3,670 (22.8)	3,761 (22.9)	91 (25)	97 (25.6)
**Height at the baseline survey**[a]										
Q1, *n* (%)	180 (42.5)	181 (42.5)	2,101 (36.7)	2,117 (36.7)	7,310 (25.6)	7,344 (25.6)	1,784 (10.9)	1,794 (10.9)	18 (4.8)	18 (4.8)
Q2, *n* (%)	143 (33.7)	144 (33.7)	1,779 (31.1)	1,790 (31.0)	9,261 (32.4)	9,292 (32.4)	4,076 (24.9)	4,089 (24.9)	51 (13.5)	51 (13.5)
Q3, *n* (%)	62 (14.6)	62 (14.6)	1,108 (19.3)	1,114 (19.3)	6,847 (24)	6,869 (23.9)	4,647 (28.4)	4,660 (28.4)	110 (29.2)	110 (29.2)
Q4, *n* (%)	39 (9.2)	39 (9.2)	740 (12.9)	744 (12.9)	5,170 (18.1)	5,185 (18.1)	5,845 (35.7)	5,858 (35.7)	198 (52.5)	198 (52.5)
**Passive smoking around 10 years old**										
Almost none, *n* (%)	185 (50.6)	218 (51.1)	2,670 (50.6)	2,936 (50.9)	13,757 (51.7)	14,908 (52.0)	7,385 (47.4)	7,813 (47.6)	160 (44.2)	168 (44.6)
1–3 times a month, *n* (%)	8 (2.2)	10 (2.4)	225 (4.3)	246 (4.3)	1,154 (4.3)	1,241 (4.3)	693 (4.4)	726 (4.4)	23 (6.4)	23 (6.2)
1–4 times a week, *n* (%)	33 (9)	38 (8.9)	378 (7.2)	420 (7.3)	2,297 (8.6)	2,466 (8.6)	1,424 (9.1)	1,492 (9.1)	30 (8.3)	31 (8.2)
Almost every day, *n* (%)	140 (38.3)	160 (37.5)	2,006 (38)	2,162 (37.5)	9,428 (35.4)	10,076 (35.1)	6,094 (39.1)	6,370 (38.8)	149 (41.2)	155 (41.1)
**Educational attainment**										
Junior high school, *n* (%)	131 (31.6)	135 (31.6)	1,204 (21.3)	1,238 (21.5)	4,996 (17.7)	5,108 (17.8)	1,299 (8)	1,324 (8.1)	28 (7.5)	46 (7.5)
High school, *n* (%)	198 (47.7)	204 (47.9)	2,916 (51.6)	2,976 (51.6)	14,925 (52.9)	15,173 (52.9)	8,256 (50.9)	8,344 (50.9)	170 (45.6)	47 (45.7)
Junior college/specialty/ college or university dropout, *n* (%)	69 (16.6)	70 (16.5)	1,278 (22.6)	1,293 (22.4)	6,891 (24.4)	6,976 (24.3)	5,312 (32.7)	5,355 (32.7)	135 (36.2)	48 (36.2)
College/university/ graduate school, *n* (%)	17 (4.1)	17 (4.0)	254 (4.5)	257 (4.5)	1,415 (5)	1,432 (5.0)	1,368 (8.4)	1,378 (8.4)	40 (10.7)	49 (10.6)

CC, complete cases; BMI, body mass index; Q, quartile.

[a] Range of height in cm for each quartile: Q1 [125,150], Q2 [151, 155], Q3 [156, 159], Q4 [160, 195].

[b] Numbers in parenthesis are percentages among total population for each column.

eTable 3. Adjusted prevalence ratio in overall population (n=95,737), in male population (n=44,079) and female population (n=51,658) — imputed dataset

		Birth weight[g]				
		<1,500 g aPR [95% CI]	1,500–2,499 g aPR [95% CI]	2,500–2,999 g aPR [95% CI]	3,000–3,999 g	≥4,000 g aPR [95% CI]
**Cardiovascular disease**						
Pooled[f]	Model 1[a]	1.85[1.47–2.32]***	1.28 [1.16–1.42]***	1.08 [1.00–1.16]	ref	0.98 [0.64–1.50]
	Model 2[b]	1.80 [1.44–2.25]***	1.27 [1.14–1.40]***	1.07 [0.99–1.16]	ref	0.99 [0.65–1.51]
	Model 3[c]	1.77 [1.42–2.22]***	1.26 [1.14–1.40]***	1.07 [0.99–1.16]	ref	0.99 [0.65–1.51]
	Model 4[d]	1.77 [1.41–2.22]***	1.27 [1.15–1.41]***	1.08 [1.00–1.17]*	ref	0.97 [0.64–1.48]
Male	Model 1[a]	1.78 [1.36–2.34]***	1.18 [1.04–1.34]**	1.05 [0.96–1.14]	ref	1.10 [0.70–1.73]
	Model 2[b]	1.74 [1.33–2.28]***	1.16 [1.02–1.32]*	1.04 [0.95–1.14]	ref	1.11 [0.70–1.74]
	Model 3[c]	1.73 [1.32–2.27]***	1.16 [1.03–1.32]*	1.04 [0.95–1.14]	ref	1.11 [0.71–1.75]
	Model 4[d]	1.74 [1.32–2.28]***	1.18 [1.04–1.34]*	1.05 [0.96–1.15]	ref	1.09 [0.69–1.71]
Female	Model 1[a]	1.98 [1.32–2.98]**	1.51 [1.27–1.80]***	1.15 [1.01–1.32]*	ref	0.58 [0.19–1.79]
	Model 2[b]	1.90 [1.28–2.84]**	1.49 [1.24–1.77]***	1.14 [0.99–1.31]	ref	0.57 [0.18–1.76]
	Model 3[c]	1.82 [1.22–2.71]**	1.47 [1.23–1.75]***	1.14 [1.00–1.31]	ref	0.56 [0.18–1.74]
	Model 4[d]	1.81 [1.21–2.69]**	1.47 [1.23–1.75]***	1.15 [1.00–1.32]*	ref	0.56 [0.18–1.73]
**Hypertension**						
Pooled[f]	Model 1[a]	1.25 [1.15–1.37]***	1.09 [1.05–1.13]***	1.05 [1.02–1.08]***	ref	0.97 [0.83–1.12]
	Model 2[b]	1.27 [1.16–1.38]***	1.08 [1.04–1.11]***	1.05 [1.03–1.08]***	ref	0.95 [0.83–1.10]
	Model 3[c]	1.26 [1.16–1.38]***	1.08 [1.04–1.11]***	1.05 [1.03–1.08]***	ref	0.96 [0.83–1.10]
	Model 4[d]	1.28 [1.18–1.40]***	1.10 [1.06–1.14]***	1.07 [1.04–1.09]***	ref	0.94 [0.81–1.08]
Male	Model 1[a]	1.15 [1.02–1.30]*	1.04 [0.99–1.09]	1.03 [0.99–1.06]	ref	1.00 [0.84–1.18]
	Model 2[b]	1.19 [1.05–1.34]**	1.04 [1.00–1.09]	1.04 [1.01–1.08]*	ref	0.98 [0.83–1.16]
	Model 3[c]	1.19 [1.05–1.34]**	1.04 [1.00–1.10]	1.04 [1.01–1.07]*	ref	0.98 [0.83–1.16]
	Model 4[d]	1.20 [1.06–1.35]**	1.07 [1.02–1.12]**	1.05 [1.02–1.09]**	ref	0.96 [0.81–1.13]
Female	Model 1[a]	1.37 [1.20–1.56]***	1.14 [1.08–1.20]***	1.08 [1.04–1.12]***	ref	0.83 [0.61–1.13]
	Model 2[b]	1.34 [1.18–1.53]***	1.10 [1.04–1.16]***	1.06 [1.02–1.10]**	ref	0.82 [0.61–1.10]
	Model 3[c]	1.34 [1.18–1.52]***	1.10 [1.04–1.16]***	1.06 [1.02–1.10]**	ref	0.82 [0.61–1.10]
	Model 4[d]	1.37 [1.21–1.56]***	1.12 [1.06–1.19]***	1.07 [1.03–1.12]***	ref	0.81 [0.60–1.09]
**Diabetes**						
Pooled[f]	Model 1[a]	1.68 [1.41–2.00]***	1.26 [1.17–1.36]***	1.08 [1.02–1.14]**	ref	1.11 [0.84–1.46]
	Model 2[b]	1.57 [1.33–1.87]***	1.24 [1.15–1.33]***	1.08 [1.02–1.14]**	ref	1.00 [0.77–1.31]
	Model 3[c]	1.57 [1.32–1.86]***	1.24 [1.15–1.33]***	1.08 [1.02–1.14]**	ref	1.01 [0.77–1.31]
	Model 4[d]	1.61 [1.36–1.92]***	1.29 [1.20–1.39]***	1.11 [1.05–1.17]***	ref	0.96 [0.73–1.25]
Male	Model 1[a]	1.55 [1.25–1.91]***	1.21 [1.10–1.32]***	1.06 [0.99–1.13]	ref	1.00 [0.72–1.38]
	Model 2[b]	1.48 [1.20–1.83]***	1.20 [1.09–1.31]***	1.07 [1.00–1.14]	ref	0.91 [0.66–1.24]
	Model 3[c]	1.49 [1.21–1.84]***	1.20 [1.09–1.31]***	1.06 [1.00–1.13]	ref	0.91 [0.67–1.24]
	Model 4[d]	1.50 [1.22–1.86]***	1.24 [1.14–1.36]***	1.09 [1.02–1.16]**	ref	0.85 [0.62–1.17]
Female	Model 1[a]	1.94 [1.45–2.60]***	1.35 [1.19–1.54]***	1.12 [1.01–1.23]*	ref	1.42 [0.84–2.40]
	Model 2[b]	1.71 [1.28–2.30]***	1.29 [1.14–1.47]***	1.09 [0.99–1.21]	ref	1.28 [0.77–2.13]
	Model 3[c]	1.68 [1.25–2.26]***	1.29 [1.13–1.47]***	1.09 [0.99–1.21]	ref	1.27 [0.76–2.12]
	Model 4[d]	1.79 [1.34–2.40]***	1.35 [1.18–1.53]***	1.13 [1.03–1.25]*	ref	1.26 [0.76–2.11]
**Hyperlipidemia**						
Pooled[f]	Model 1[a]	0.98 [0.86–1.12]	1.04 [0.99–1.08]	1.03 [1.00–1.06]	ref	1.04 [0.88–1.23]
	Model 2[b]	0.99 [0.86–1.12]	1.02 [0.98–1.07]	1.02 [0.99–1.06]	ref	1.04 [0.88–1.24]
	Model 3[c]	0.99 [0.87–1.13]	1.02 [0.98–1.07]	1.02 [0.99–1.06]	ref	1.04 [0.88–1.23]
	Model 4[d]	0.99 [0.87–1.13]	1.03 [0.99–1.08]	1.03 [1.00–1.06]	ref	1.04 [0.87–1.23]
Male	Model 1[a]	0.88 [0.71–1.09]	0.97 [0.91–1.04]	0.98 [0.94–1.03]	ref	1.01 [0.82–1.25]
	Model 2[b]	0.92 [0.75–1.14]	0.97 [0.91–1.05]	0.99 [0.95–1.04]	ref	1.01 [0.82–1.25]
	Model 3[c]	0.92 [0.74–1.13]	0.97 [0.91–1.05]	0.99 [0.95–1.04]	ref	1.01 [0.82–1.25]
	Model 4[d]	0.92 [0.75–1.14]	0.98 [0.92–1.06]	1.00 [0.95–1.05]	ref	1.00 [0.81–1.24]
Female	Model 1[a]	1.04 [0.88–1.22]	1.07 [1.01–1.13]*	1.05 [1.01–1.09]*	ref	0.98 [0.75–1.30]
	Model 2[b]	1.02 [0.86–1.20]	1.05 [0.99–1.11]	1.04 [0.99–1.08]	ref	0.99 [0.75–1.31]
	Model 3[c]	1.02 [0.86–1.21]	1.05 [0.99–1.11]	1.04 [0.99–1.08]	ref	0.99 [0.75–1.31]
	Model 4[d]	1.02 [0.86–1.20]	1.05 [0.99–1.11]	1.04 [1.00–1.08]	ref	0.99 [0.75–1.31]
**Gout**						
Pooled[f]	Model 1[a]	0.92 [0.67–1.26]	0.96 [0.87–1.07]	0.98 [0.91–1.05]	ref	0.92 [0.66–1.27]
	Model 2[b]	1.04 [0.76–1.42]	1.03 [0.93–1.15]	1.03 [0.96–1.10]	ref	0.89 [0.64–1.23]
	Model 3[c]	1.04 [0.76–1.42]	1.03 [0.93–1.15]	1.03 [0.96–1.10]	ref	0.89 [0.64–1.23]
	Model 4[d]	1.05 [0.77–1.44]	1.06 [0.95–1.19]	1.05 [0.98–1.12]	ref	0.85 [0.61–1.18]
Male	Model 1[a]	0.88 [0.63–1.22]	0.94 [0.84–1.05]	0.97 [0.91–1.04]	ref	0.93 [0.67–1.29]
	Model 2[b]	0.99 [0.71–1.37]	1.00 [0.90–1.12]	1.02 [0.95–1.10]	ref	0.90 [0.65–1.25]
	Model 3[c]	0.99 [0.71–1.37]	1.00 [0.90–1.12]	1.02 [0.95–1.10]	ref	0.90 [0.65–1.25]
	Model 4[d]	1.00 [0.73–1.39]	1.04 [0.93–1.16]	1.04 [0.97–1.12]	ref	0.86 [0.62–1.20]
Female	Model 1[a]	1.93 [0.60–6.21]	1.52 [0.93–2.49]	1.10 [0.76–1.61]	ref	e
	Model 2[b]	2.12 [0.66–6.82]	1.66 [1.00–2.75]*	1.17 [0.80–1.71]	ref	e
	Model 3[c]	1.98 [0.61–6.38]	1.63 [0.99–2.70]	1.17 [0.80–1.72]	ref	e
	Model 4[d]	1.95 [0.60–6.30]	1.61 [0.97–2.70]	1.16 [0.79–1.72]	ref	e

aPR, adjusted prevalence ratio; BMI, body mass index; CI, confidence interval; ref, reference.

[a] Model 1: age + birth year + gender for pooled analysis, age + birth year for gender specific analysis.

[b] Model 2: model 1 + educational attainment, family history, passive smoking around 10 years old, height at the baseline study, having elder siblings.

[c] Model 3: model 2 + smoking status.

[d] Model 4: model 3 + BMI at 20 years old.

[e] aPR in birth weight category 4 kg and above were not calculatable among females for gout as there were no cases of gout in this group.

[f] Interaction between gender and birth weight (p<0.05) was observed for hypertension, diabetes, hyperlipidemia and gout but not for cardiovascular disease.

[g] *P* -values: * P <0.05, ** P <0.01, *** P <0.001.

eTable 4. Adjusted prevalence ratio in overall population (n=88,653), male population (n=41,156) and female population (n=47,497) on medication — complete cases dataset

		Birth weight[e]				
		<1,500 g aPR [95% CI]	1,500–2,499 g aPR [95% CI]	2,500–2,999 g aPR [95% CI]	3,000–3,999 g	≥4,000 g aPR [95% CI]
**Hypertension (on medication)**						
Pooled[d]	Model 1[a]	1.27 [1.14–1.41]***	1.10 [1.06–1.15]***	1.06 [1.03–1.09]***	ref	1.00 [0.86–1.18]
	Model 2[b]	1.29 [1.17–1.43]***	1.08 [1.04–1.13]***	1.06 [1.03–1.09]***	ref	0.99 [0.85–1.16]
Male	Model 1[a]	1.14 [0.99–1.32]	1.04 [0.99–1.10]	1.04 [1.00–1.07]	ref	1.01 [0.84–1.22]
	Model 2[b]	1.19 [1.04–1.37]*	1.04 [0.99–1.09]	1.05 [1.01–1.09]**	ref	0.99 [0.83–1.18]
Female	Model 1[a]	1.40 [1.21–1.63]***	1.17 [1.10–1.24]***	1.09 [1.04–1.14]***	ref	0.92 [0.67–1.26]
	Model 2[b]	1.38 [1.19–1.60]***	1.13 [1.06–1.19]***	1.07 [1.02–1.12]**	ref	0.92 [0.67–1.25]
**Diabetes (on medication)**					ref	
Pooled[d]	Model 1[a]	1.66 [1.34–2.06]***	1.25 [1.15–1.36]***	1.07 [1.01–1.14]*	ref	0.88 [0.61–1.26]
	Model 2[b]	1.57 [1.27–1.95]***	1.23 [1.12–1.34]***	1.07 [1.01–1.14]*	ref	0.80 [0.56–1.14]
Male	Model 1[a]	1.53 [1.18–1.99]**	1.14 [1.02–1.27]*	1.03 [0.95–1.11]	ref	0.82 [0.54–1.25]
	Model 2[b]	1.50 [1.16–1.95]**	1.12 [1.01–1.25]*	1.03 [0.96–1.11]	ref	0.75 [0.50–1.13]
Female	Model 1[a]	1.94 [1.34–2.80]***	1.49 [1.28–1.73]***	1.18 [1.05–1.32]**	ref	1.01 [0.48–2.11]
	Model 2[b]	1.67 [1.16–2.42]**	1.43 [1.23–1.67]***	1.15 [1.03–1.29]*	ref	0.88 [0.43–1.81]
**Hyperlipidemia (on medication)**					ref	
Pooled[d]	Model 1[a]	0.96 [0.81–1.15]	1.07 [1.02–1.13]*	1.04 [1.00–1.08]*	ref	1.04 [0.84–1.29]
	Model 2[b]	0.96 [0.81–1.14]	1.05 [1.00–1.11]	1.03 [0.99–1.07]	ref	1.05 [0.84–1.30]
Male	Model 1[a]	0.94 [0.71–1.24]	1.02 [0.93–1.11]	0.99 [0.93–1.05]	ref	1.03 [0.78–1.36]
	Model 2[b]	0.98 [0.75–1.29]	1.02 [0.93–1.11]	1.00 [0.95–1.07]	ref	1.02 [0.77–1.35]
Female	Model 1[a]	0.96 [0.77–1.20]	1.10 [1.03–1.18]**	1.07 [1.01–1.12]*	ref	0.95 [0.67–1.35]
	Model 2[b]	0.93 [0.74–1.16]	1.06 [0.99–1.14]	1.04 [0.99–1.10]	ref	0.97 [0.68–1.37]
**Gout (on medication)**					ref	
Pooled[d]	Model 1[a]	0.91 [0.61–1.34]	0.95 [0.84–1.08]	0.96 [0.89–1.04]	ref	0.90 [0.61–1.34]
	Model 2[b]	1.02 [0.69–1.51]	1.02 [0.89–1.16]	1.01 [0.93–1.10]	ref	0.87 [0.59–1.28]
Male	Model 1[a]	0.94 [0.63–1.39]	0.94 [0.82–1.07]	0.96 [0.89–1.05]	ref	0.91 [0.62–1.35]
	Model 2[b]	1.05 [0.71–1.56]	1.00 [0.88–1.14]	1.01 [0.93–1.10]	ref	0.88 [0.59–1.30]
Female	Model 1[a]	-	1.26 [0.66–2.42]	0.88 [0.54–1.44]	ref	[c]
	Model 2[b]	-	1.43 [0.73–2.80]	0.95 [0.58–1.56]	ref	[c]

aPR, adjusted prevalence ratio; CI, confidence interval; ref, reference.

[a] Model 1: age + birth year + gender for pooled analysis, age + birth year for gender specific analysis.

[b] Model 2: model 1 + educational attainment, family history, passive smoking around 10 years old, height at the baseline study, having elder eiblings.

[c] aPR in birth weight category 4 kg and above were not calculatable among females for gout as there were no cases of gout in this group.

[d] Interaction between gender and birth weight was not observed.

[e] *P* -values: * *P* <0.05, ** *P* <0.01, *** *P* <0.001.

eTable 5. Adjusted prevalence ratio in overall population (n=88,653), male population (n=41,156) and female population (n=47,497) ー including and excluding angina

		Birth weight[d]				
		<1,500 g aPR [95% CI]	1,500–2,499 g aPR [95% CI]	2,500–2,999 g aPR [95% CI]	3,000–3,999 g	≥4,000 g aPR [95% CI]
**Cardiovascular disease**						
Pooled[c]	Model 1[a]	1.82 [1.42–2.35]***	1.27 [1.14–1.41]***	1.08 [1.00–1.17]	ref	1.03 [0.67–1.58]
	Model 2[b]	1.76 [1.37–2.26]***	1.25 [1.12–1.39]***	1.07 [0.99–1.16]	ref	1.03 [0.67–1.59]
Male	Model 1[a]	1.72 [1.26–2.34]**	1.18 [1.03–1.34]*	1.07 [0.97–1.17]	ref	1.14 [0.71–1.82]
	Model 2[b]	1.68 [1.23–2.28]**	1.16 [1.01–1.32]*	1.06 [0.97–1.17]	ref	1.15 [0.72–1.84]
Female	Model 1[a]	2.07 [1.32–3.22]**	1.48 [1.23–1.78]***	1.12 [0.97–1.29]	ref	0.64 [0.21–1.98]
	Model 2[b]	1.96 [1.27–3.03]**	1.45 [1.20–1.75]***	1.10 [0.95–1.27]	ref	0.62 [0.20–1.92]
**Cardiovascular disease excluded angina**						
Pooled[c]	Model 1[a]	1.90 [1.42–2.54]***	1.24 [1.09–1.41]**	1.08 [0.99–1.19]	ref	1.09 [0.67–1.78]
	Model 2[b]	1.80 [1.35–2.41]***	1.22 [1.07–1.39]**	1.07 [0.98–1.18]	ref	1.10 [0.68–1.79]
Male	Model 1[a]	1.87 [1.32–2.63]***	1.18 [1.01–1.38]*	1.09 [0.98–1.21]	ref	1.24 [0.74–2.09]
	Model 2[b]	1.78 [1.27–2.51]**	1.15 [0.98–1.35]	1.08 [0.97–1.20]	ref	1.26 [0.75–2.12]
Female	Model 1[a]	1.98 [1.13–3.46]*	1.41 [1.12–1.78]**	1.08 [0.91–1.29]	ref	0.59 [0.15–2.37]
	Model 2[b]	1.89 [1.09–3.27]*	1.38 [1.09–1.75]**	1.07 [0.89–1.27]	ref	0.57 [0.14–2.28]

aPR, adjusted prevalence ratio; CI, confidence interval; ref, reference.

[a] Model 1: age + birth year + gender for pooled analysis, age + birth year for gender specific analysis.

[b] Model 2: model 1 + educational attainment, family history, passive smoking around 10 years old, height at the baseline study, having elder siblings.

[c]Interaction between gender and birth weight was not observed.

[d]*P* -values: * *P* <0.05, ** *P* <0.01, *** *P* <0.001.

eFigure 1. Population flow chart

114,105 participants who answered the baseline questionnaire

→ 18,368 participants with no data on birth weight

↓

95,737 participants with data on birth weight (population for sensitivity analysis 1)

multiple imputation used to impute missing data on covariates

→ 7,084 participants with missing data on any covariates

↓

88,653 participants with data on all covariates (complete case dataset)
